# Does Particulate Matter Modify the Short-Term Association between Heat Waves and Hospital Admissions for Cardiovascular Diseases in Greater Sydney, Australia?

**DOI:** 10.3390/ijerph16183270

**Published:** 2019-09-05

**Authors:** Marissa Parry, Donna Green, Ying Zhang, Andrew Hayen

**Affiliations:** 1 Climate Change Research Centre, University of New South Wales, Sydney, NSW 2052, Australia; 2 ARC Centre of Excellence for Climate Extremes, University of New South Wales, Sydney, NSW 2052, Australia; 3 Australia and NHMRC Centre for Air Pollution, Energy and Health Research, University of Sydney, Sydney, NSW 2052, Australia; 4 School of Public Health, University of Sydney, Sydney, NSW 2006, Australia; 5 Faculty of Health, University of Technology Sydney, Sydney, NSW 2007, Australia

**Keywords:** heat waves, particulate matter, Sydney, Australia, hospital admissions, cardiovascular disease

## Abstract

Little is known about the potential interactive effects of heat waves and ambient particulate matter on cardiovascular morbidity. A time-stratified case-crossover design was used to examine whether particulate matter (PM_10_) modifies the association between heat waves and emergency hospital admissions for six cardiovascular diseases in Greater Sydney, Australia during the warm season for 2001–2013. We estimated and compared the effect of heat waves on high- and low-level PM_10_ days at lag_0_–lag_2_, adjusting for dew-point temperature, ambient ozone, ambient nitrogen dioxide, and public holidays. We also investigated the susceptibility of both younger (0–64 years) and older populations (65 years and above), and tested the sensitivity of three heat wave definitions. Stronger heat wave effects were observed on high- compared to low-level PM_10_ days for emergency hospital admissions for cardiac arrest for all ages combined, 0–64 years and 65 years and above; conduction disorders for 0–64 years; and hypertensive diseases for all ages combined and 0–64 years. Overall, we found some evidence to suggest that PM_10_ may modify the association between heat waves and hospital admissions for certain cardiovascular diseases, although our findings largely differed across disease, age group, lag, and heat wave definition.

## 1. Introduction

Cardiovascular disease is a major cause of death both worldwide and in Australia [1,2]. Some studies have shown that high temperatures and heat waves are associated with increased risk of hospitalisation for cardiovascular diseases generally [3,4,5], and specific cardiovascular diseases including ischemic heart disease and cardiac (or heart) dysrhythmias [3,4,6]. Elevated temperature and heat waves have also been shown to be associated with an increased risk of out-of-hospital cardiac arrest [7]. A short lag effect has been observed, with positive associations between high temperatures and hospitalisations for cardiovascular diseases reported on the same day of exposure [4] and between 1–3 days after exposure [3]. Other studies, however, including two meta-analyses, have reported null or negative associations between high temperatures and hospital admissions for cardiovascular diseases [8,9,10,11], but Phung et al. [10] reported a small, positive heat wave effect.

Ambient particulate matter with an aerodynamic diameter less than 10 μm, known as particulate matter (PM_10_), is comprised of both solid particles and liquid droplets from natural and anthropogenic sources [12]. Levels and mixtures of PM_10_ can depend on season and temperature, with bushfire smoke and dust storms being important sources during the warm season in Australia, and wood heaters an important source in the cool season [13]. Studies have shown that elevated levels of PM_10_ are associated with an increased risk of hospitalisation for all cardiovascular or cardiac diseases [14,15,16] and specific diseases including ischemic heart disease [17,18], heart failure [19], and heart arrhythmias and conduction disorders [15], particularly among the elderly. Elevated levels of PM_10_ have also been shown to be associated with an increased risk of out-of-hospital cardiac arrest [20]. A few studies have assessed, or controlled for, the potential confounding effects of PM_10_ when estimating the association between extreme heat and hospitalisations for cardiovascular diseases (e.g., [21,22]). 

Little is known about the potential joint or interactive effects of high temperatures, particularly heat waves, and PM_10_ on cardiovascular health outcomes. This is concerning given that the joint effect of weather and air pollution on health outcomes is thought to be greater than the risk derived from the individual impacts of these two exposures [23]. There is also some suggestion that an interactive effect between air pollution and temperature may be biologically plausible [24]. Some studies from Europe and Asia have investigated whether temperature modifies the association between PM_10_ and all-cause and/or cardiovascular mortality [25,26,27,28,29,30]. Most of these studies have generally found stronger associations at high compared to moderate or low level temperatures, although such evidence of effect modification has not been consistently statistically significant. However, Cheng and Kan [28] found a statistically significant interaction between low, but not high, temperature and PM_10_ on total and cardiovascular mortality in Shanghai, China.

Few studies have investigated whether PM_10_ modifies the association between high temperatures, particularly heat waves, and cardiovascular health outcomes. Some have found stronger associations between high temperatures or heat waves and all-cause and/or cardiovascular mortality at higher levels of PM_10_, although not all have reported evidence of statistical significance [30,31,32,33]. Other studies have found no evidence of an interaction between temperature and PM_10_ on mortality [34,35]. Little work, however, has examined whether PM_10_ modifies the association between temperature or heat waves and cardiovascular morbidity, particularly cause-specific cardiovascular morbidity. One Australian study found that PM_10_ modified the association between temperature and cardiovascular hospital admissions at different lags in Brisbane, however it found little evidence of effect modification for cardiovascular emergency presentations [36]. Further, a recent Korean study found no evidence of a significant interactive effect between heat waves and PM_10_ on out-of-hospital cardiac arrest [7]. 

The frequency, intensity, and duration of heat waves is expected to increase in the future under climate change across most land areas globally, including Australia [37,38]. It is therefore important to clarify and enhance our understanding of the association between heat waves and cardiovascular morbidity to inform climate change adaptation planning in the health sector. This study aimed to examine whether PM_10_ modifies the short-term association between heat waves and hospital admissions for specific cardiovascular diseases in Greater Sydney, Australia. We investigated the susceptibility of both younger (0–64 years) and older populations (65 years and above), and tested the sensitivity of three heat wave definitions. 

## 2. Materials and Methods 

### 2.1. Meteorological Data 

Daily weather data for all stations located in the Sydney Statistical Division (SSD) with near complete coverage of the period of 2001 to 2013 were obtained from the Australian Government’s Bureau of Meteorology (*n* = 17). Before identifying extreme temperature events in a climate time series, such as summer heat waves, it is important that the data undergo quality control checks [39]. This is because it is possible for incorrect data entries to be considered as real “extreme” values and included in further analyses [39]. To ensure our observational weather data was of the highest possible quality, we performed a series of quality control checks on the observed daily maximum, minimum, and dew-point temperature values for each weather station, and also tested for inhomogeneities in each daily maximum and minimum time series to inspect their overall quality. High quality stations (*n* = 15) were then used to calculate the respective city-wide averages for each temperature metric if they had a total missing value count of ≤2.5% of the study period. The missing value threshold was set at ≤2.5% to maximise the number of stations included in the calculation of the average and subsequent spatial coverage of the SSD, while also ensuring that the quality of those stations that were included remained high. The daily average mean temperature was calculated as the mean of the city-wide daily average maximum and minimum temperature values. For dew-point temperature, as the observations were recorded at 3-hour intervals over a 24-hour period, the city-wide average value for each time interval was first calculated with those stations where the missing value count was ≤2.5% of the study period, then the overall 24-hour daily average was calculated from these averaged time interval values. 

In the absence of a standard heat wave definition, we selected and compared three heat wave definitions for this study. Previous studies have shown that the choice of heat wave definition can alter the magnitude and statistical significance of the association between heat waves and adverse health outcomes [40,41]. We defined a heat wave as two or more consecutive days where the temperature metric (three temperature metrics were selected and compared: maximum temperature (HWD01), mean temperature (HWD02), and minimum temperature (HWD03)) is greater than or equal to the 90th percentile of the warm season (1 November to 31 March) during 2001 to 2013. We compared heat wave definitions with alternative temperature metrics, rather than temperature thresholds or durations, to ensure that we kept an adequate number of heat wave days to conduct the analysis. 

### 2.2. Ambient Air Pollution Data 

Daily ambient air pollution data for all stations located in the SSD were obtained from the NSW Office of Environment and Heritage for 2001 to 2013. Daily data were obtained for the following air pollutants and used in this study: ozone (1 h average 24 h maximum value (pphm)); nitrogen dioxide (1 h average 24 h maximum value (pphm)), and particulate matter (particles with an aerodynamic diameter of less than 10 μm, PM_10_) (1 h average 24 h average value). The NSW Office of Environment and Heritage follows several quality assurance procedures to ensure the data are precise, accurate, representative, and comparable [42]. Negative daily values were assigned a value of 0. Stations that had a missing value count of ≤5% of the study period were used to calculate the daily city-wide average for each pollutant. Junger and Ponce de Leon [43] regarded a missing data level of 5% as the best-case scenario in their application of time-series air pollution data. Similar to the threshold selection for our meteorological data, a threshold of 5% was optimal in allowing us to maximise the number of stations included in the calculation of the average and subsequent spatial coverage of the SSD, while also ensuring that the quality of those stations that were included remained high. PM_2.5_ (ambient particulate matter with an aerodynamic diameter less than 2.5 μm) was not considered in this study given the smaller spatial and temporal coverage of the data across the Greater Sydney region. 

### 2.3. Health Data 

Individual-level daily hospital admission records with a principal diagnosis of I00-I99 (ICD-10-AM) for all public and private hospitals located in the SSD were obtained from the NSW Ministry of Health, Admitted Patient Data Collection, for 2001 to 2013 as part of a larger dataset (*n =* 1,570,805). All exact duplicate records were extracted and removed (*n =* 1,570,741, 64 records removed), as well those records with an admission date outside of 1 July 2001–30 June 2013 (*n =* 1,499,661, 71,080 records removed). Records that were classified as “emergency” hospital admissions (EHAs) were then selected for analysis to eliminate “pre-planned” hospital admissions (*n* = 1,132,737, records removed 366924) [44]. We then extracted and removed remaining records with an implausible, unknown, or missing entry for age (ranged deemed plausible: 0–115 years) or sex (required entry: male or female) (*n* = 1,132,705, records removed 32). Those records with a principal diagnosis of ischemic heart disease (ICD-10-AM: I20-I25), heart failure (ICD-10-AM: I50), cardiac arrest (ICD-10-AM: I46), heart arrhythmia (ICD-10-AM: 147-I49), conduction disorders (ICD-10-AM: I44-I45), and hypertensive diseases (ICD-10-AM: I10-I15) were then selected and aggregated into daily counts. To investigate the susceptibility of both younger and older populations, we stratified the data into two age groups: 0–64 years and 65 years and above. 

### 2.4. Study Design and Statistical Analysis 

We used a time-stratified case-crossover study design [45,46]. This design has been used in previous studies to estimate the association between heat waves and hospital admissions [47,48], and has been shown to produce similar results to the alternate time-series design [49]. The design is equivalent to a matched pair case-control design: it compares a case’s exposure on the day of an adverse health event (e.g., hospital admission) to their exposure on control days (or referent times), which are selected before and/or after the event [46,50,51]. Since each case acts as their own control, personal characteristics such as sex and smoking status are controlled for by matching [51]. We used the time-stratified approach to select control days to avoid potential bias introduced by other approaches, such as the unidirectional and bidirectional designs [46]. We matched cases and controls on day of the week and within the same month, and thus controlled for the confounding effects of season and long-term trends by design.

We used conditional logistic regression to estimate the association between heat waves and EHAs for our six selected cardiovascular diseases. We first estimated the association with and without adjusting for daily average PM_10_ at lag_0_. All of the models included daily average dew-point temperature, daily average nitrogen dioxide, daily average ozone, and public holidays as covariates. More specifically, we adjusted for daily average dew-point temperature [52] using a natural cubic spline (df = 3, knots at quantiles), daily average nitrogen dioxide (1 h average 24 h maximum value (pphm)), daily average ozone (1 h maximum 24 h average value (pphm)), and public holidays. To determine the most appropriate way to model dew-point temperature, we conducted sensitivity tests modelling this variable as a natural cubic spline with 3 and 2 degrees of freedom, and as a linear variable at lag_0_. As the coefficients of the heat wave effect were largely similar across the three modelling approaches, we selected to model dew-point temperature as a natural cubic spline with 3 degrees of freedom to be consistent with previous work [53]. 

To examine whether PM_10_ modifies the association between heat waves and EHAs for our six selected cardiovascular diseases, we estimated and compared heat wave effects on days with high and low levels of PM_10_ at lag_0_-lag_2._ High and low level PM_10_ days were defined as those where the daily average PM_10_ value was ≥90th and <90th percentile of the warm season during 2001 to 2013, respectively (Note: 90th percentile of the distribution was equal to 30.52 µg/m^3^). We created an interaction term between high and low level PM_10_ days (1 = high, 0 = low) and heat wave days (1 = yes, 0 = no). This term was added to the model, along with the respective individual variables and potential confounding variables described in the previous paragraph. We selected the threshold of the 90th percentile for two main reasons: to ensure there was a reasonably equal distribution of high and low level PM_10_ days across heat wave days for the three definitions for a fair comparison and to compare and estimate heat wave effects on days with the more extreme values of PM_10_. 

The statistical analyses were conducted in the “R” Statistical Computing Environment (Version 3.2.1) using the “season” and “dlnm” packages. As we wanted to examine the impact of summer heat waves, we restricted our analyses to the warm season (1 November to 31 March) for 2001 to 2013. The effects are presented as odds ratio with their corresponding 95% confidence intervals. The figure is presented on the log scale. A *p*-value of <0.05 was considered significant. 

This project was approved by the University of New South Wales Human Research Low Risk Ethics Advisory Committee Panel H. 

## 3. Results

Descriptive statistics for selected weather and ambient air pollution variables during the study period are presented in Table 1. The mean daily average maximum temperature was 26.0 °C, and the mean daily average value of PM_10_ was 20.43 µg/m^3^. 

Table 2 shows descriptive statistics for selected EHAs for six cardiovascular diseases for all ages combined and two age groups: 0–64 years and 65 years and over. Ischemic heart disease had the highest number of total EHAs during the study period with 68,334, while cardiac arrest had the lowest with 1861. For each cardiovascular disease, the older age group had a higher number of EHAs than the younger age group.

A summary of the heat wave characteristics for each heat wave definition used is provided in Table 3. HWD03 had the highest total number of heat wave days during the study period and the longest average heat wave duration of 2.92 days. HWD02 had the highest number of total heat wave events with 43. 

**Figure 1:**
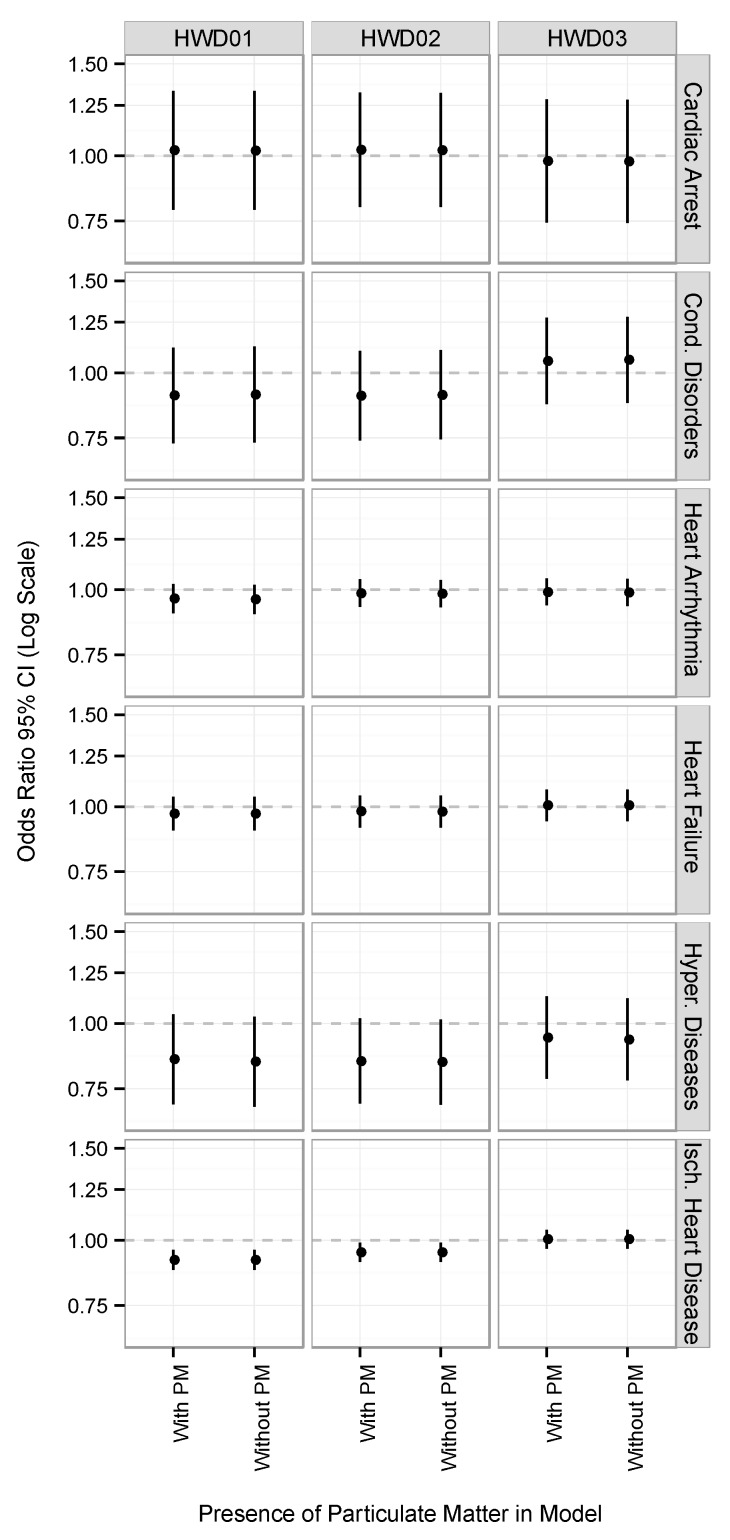
The association between heat wave days and “emergency” hospital admissions (EHAs) for six cardiovascular diseases with and without controlling for daily average particulate matter (PM_10_) at lag_0_ in the SSD during the warm season, 2001 to 2013. Note: Cond. Disorders is conduction disorders; Hyper. Diseases is hypertensive diseases; Isch. Heart Disease is Ischemic Heart Disease.

Figure 1 shows the association between heat wave days and EHAs for six cardiovascular diseases with and without controlling for daily average PM_10_ at lag_0_ for all ages. For all diseases and across the three heat wave definitions, controlling for daily average PM_10_ had little effect on the health risk estimates. Negative associations were found between heat wave days and EHAs for heart arrhythmia and hypertensive diseases for all three heat wave definitions, although these associations were not statistically significant. Negative associations were also found between heat wave days and EHAs for ischemic heart disease, heart failure, and conduction disorders for HWD01 and HWD02, and small positive associations were found for HWD03. The negative associations found for EHAs for ischemic heart disease for HWD01 and HWD02 were statistically significant. Small, positive associations were found between heat wave days and EHAs for cardiac arrest for HWD01 and HWD02, and negative associations were found for HWD03. 

Table 4 shows the association between heat wave days and EHAs for six cardiovascular diseases at two levels of PM_10_ (high: ≥90th percentile; and low: <90th percentile) for all ages at lag_0_ and lag_1_. The results for lag_2_ are presented in Table A1 in Appendix A. A positive, statistically significant interaction was found between heat wave and high-level PM_10_ days on EHAs for hypertensive diseases at lag_1_ for HWD03, meaning that there was a stronger effect on EHAS on high-level PM_10_ days than on low-level PM_10_ days. Heat wave effects were also stronger on high-level PM_10_ days for hypertensive diseases for HWD03 at lag_0_ and lag_2_, but the *p*-value of the interaction term was not statistically significant. The impact of heat waves on EHAs for cardiac arrest was generally found to be stronger on days with high levels of PM_10_ across most lags and definitions, although none of the interaction terms were statistically significant. A negative, statistically significant interaction was found between heat wave and high-level PM_10_ days on EHAs for ischemic heart disease at lag_2_ for HWD01 (meaning that there was a weaker effect on EHAS on high-level PM_10_ days than on low-level PM_10_ days), but not at lag_0_ or lag_1_.

Table 5 shows the association between heat wave days and EHAs for six cardiovascular diseases at two levels of PM_10_ (high: ≥90th percentile; and low: <90th percentile) for younger and older populations at lag_0_ and lag_1_. The results for lag_2_ are presented in Table A2 in the Appendix A. A positive, statistically significant interaction was found between heat wave and high-level PM_10_ days on EHAs for cardiac arrest in the older age group for HWD01 at lag_1_ and lag_2_, and for HWD02 at lag_1_. Heat wave effects were also found to be stronger on high-level PM_10_ days at lag_0_ for HWD02, and at lag_0_ and lag_1_ for HWD03 in the younger age group, but no evidence of a statistically significant interaction was found. The impact of heat waves on EHAs for conduction disorders was stronger on high-level PM_10_ days for all definitions and lags, and on EHAs for hypertensive diseases for HWD02 and HWD03 at all lags and lag_1_ for HWD01 in the younger population. Stronger heat wave effects on high- compared to low-level PM_10_ days were found for EHAs for heart failure at lag_1_ for HWD03 in the older age group. A negative, statistically significant interaction was found between heat wave and high-level PM_10_ days on EHAs for heart arrhythmia for HWD01 at lag_1_ in the younger age group. 

## 4. Discussion

This study examined whether PM_10_ modifies the association between heat waves and EHAs for six cardiovascular diseases in Greater Sydney, Australia. We estimated and compared the effect of heat waves on high- and low-level PM_10_ days at lag_0_–lag_2_ for three age groups: all ages combined, 0–64 years, and 65 years and above, and tested the sensitivity of three heat wave definitions. We found some evidence that PM_10_ modifies the association between heat waves and EHAs for certain cardiovascular diseases. Stronger heat wave effects were observed on high- compared to low-level PM_10_ days for EHAs for cardiac arrest for all three age groups; conduction disorders for 0–64 years; and hypertensive diseases for all ages combined and 0–64 years. These findings, however, were generally not consistent across all heat wave definitions and lags. Positive, statistically significant interactions were found only for EHAs for hypertensive diseases (all ages combined) and cardiac arrest (65 years and above). 

It is difficult to directly compare our findings to previous studies, as most of the work to date examining the potential interactive effects of temperature or heat waves and PM_10_ on cardiovascular health outcomes has considered cardiovascular mortality (e.g., [26,27,30,32,33,34]). Few studies have considered cardiovascular morbidity as the health outcome, particularly cause-specific cardiovascular morbidity [36,54,55]. Much like our findings, the results of the studies considering cardiovascular morbidity have been broadly inconsistent, although different exposure variables have been considered (i.e., temperature, season, and relative humidity). For example, Ren et al. [36] found evidence of a statistical interaction between temperature and total cardiovascular hospital admissions at different lags in Brisbane, Australia, but found no such evidence for total cardiovascular emergency presentations. Qiu et al. [55] reported that the association between PM_10_ and emergency hospital admissions for ischemic heart disease was strongest in the cool season and at lower levels of relative humidity in Hong Kong, China. Further, Kang et al. [7] found no evidence of a significant interactive effect between heat waves and PM_10_ on out-of-hospital cardiac arrest in Korea, which is in general disagreement with our findings regarding EHAs for cardiac arrest. The level and source composition of PM_10_ differs across regions and cities [56,57,58], as does population acclimatisation to temperature changes and heat waves [1,59]. It is therefore important to conduct further localised studies to account for these differences and clarify our understanding of any potential interactive effects of these environmental exposures on cardiovascular morbidity. 

It is plausible that air pollution and heat exposure may interact on a biological level, although the exact causal pathways and mechanisms involved are not known. The activation of the body’s thermoregulatory system and mechanisms during heat stress can facilitate and increase the absorption and entry of toxins and air pollutants into the body, as well as alter the body’s response to such substances [24]. The strength of the toxicity of a chemical or toxin on a biological system can be exacerbated by increased body temperature [24,60]. Passive heat exposure can stress the cardiovascular system, where increased skin blood flow during thermoregulation results in increased cardiac output, which in turn is mediated by increases in heart rate [61]. Madaniyazi et al. [62] observed a “V” shaped relationship between mean temperature and heart rate and blood pressure (systolic and diastolic) in Chinese adults, finding heat effects above certain thresholds. Others have, however, observed a decrease in systolic blood pressure with an increase in ambient temperature [63]. Ren et al. [64] found that increased ambient temperature is associated with decreased heart rate variability (HRV) during the warm season, but found no evidence of an interactive effect between ambient temperature and PM_2.5_ on HRV. Particulate matter may also adversely affect the cardiovascular system by directly entering into the systemic circulation (smaller particles: PM_2.5_ or PM_1.0_), or indirectly by affecting the autonomic nervous system or inducing an inflammatory response [65]. Stafoggia et al. [26] noted that their findings of stronger PM_10_ effects on mortality during the warm season might be a result of increased exposure to this pollutant, with individuals more likely to open their windows and spend time outdoors during the summer months. 

We observed positive, statistically significant interactions between heat wave and high-level PM_10_ days on EHAs for cardiac arrest among the elderly. Previous studies examining the susceptibility of specific age groups to the potential interactive effects of high temperatures or heat waves and PM_10_ on cardiovascular mortality have generally found effect modification to be more pronounced among the elderly [27,31,33]. The elderly are particularly susceptible to extreme heat exposure due to their decreased capacity to effectively thermoregulate, with sweat gland output, blood flow to the skin, and cardiac output reduced [66]. Given the general decline of the body’s physiological processes with age and the higher prevalence of cardiovascular diseases among older age groups, the elderly are also susceptible to the adverse effects of particulate matter [67]. We also found some evidence of effect modification in the younger age group for certain diseases. The reasons for this are unclear, although it may be because younger populations are generally more physically active than older populations [68], which may result in more time spent outdoors, subsequently increasing their exposure levels. 

We found positive, statistically significant interactions at lag_1_ and lag_2_ for certain cardiovascular diseases, but not at lag_0_. Evidence of an interactive effect between high temperature and high-levels of PM_10_ on cardiovascular health outcomes has also been found at certain lags [25,36]. For example, Qian et al. [25] observed stronger PM_10_ effects on cardiovascular mortality at high compared to normal level temperatures at lag_0–1_ in Wuhan, China. Short lag effects have also been observed when examining the independent effects of high temperatures and PM_10_ on cardiovascular morbidity [3,15]. Positive, statistically significant interactions were also found for some heat wave definitions only. The choice of heat wave definition has been shown to affect both the magnitude and statistical significance of the association between heat waves and health outcomes [69]. Each of the three heat wave definitions used in this study identified different days as “exposure” days, and the total number of exposure days varied between our definitions (See Table 3). It is likely that this affected our models, as well as the calculation of the interaction term between heat wave and high-level PM_10_ days. It is also possible that different temperature metrics (maximum, mean, minimum) may have different impacts on cardiovascular health outcomes, although differences in their interaction with PM_10_ is unclear. For example, Kang et al. [7] found that the risk of out-of-hospital cardiac arrest during heat waves was highest in the afternoon (3 p.m. to 5 p.m.), which coincided with the peak of daily outdoor temperature. 

A few negative, statistically significant interactions were found, and negative associations were observed across both high- and low-level PM_10_ days and in Figure 1 for certain cardiovascular diseases. Several previous studies have also found null or negative associations between increased temperature or extreme heat and hospital admissions for cardiovascular diseases [8,9,11]. Such findings are in contrast to the positive associations often observed between high temperature or heat waves and cardiovascular mortality across several regions, particularly among the elderly [70,71]. The exact reasons for the differences found between these cardiovascular health outcomes are not known. One possible explanation is that individuals may die quickly from cardiovascular disease during high temperatures before they are able to seek medical attention or be admitted to hospital [72]. 

This study has some potential strengths. To the best of our knowledge, this is the first study to examine the potential interactive effects of heat waves and PM_10_ on cause-specific cardiovascular hospital admissions in an Australian city. By examining and comparing six specific cardiovascular diseases, we have shown that some conditions may be more susceptible to the potential interactive effects of heat waves and PM_10_ than others (e.g., cardiac arrest). We also analysed a relatively long period of time series data (12 years) and controlled for other ambient air pollutants including ozone and nitrogen dioxide. 

This study has some potential limitations. The analysis was performed for a single city and, therefore, our results may not be generalisable given that PM_10_ levels and mixtures can vary geographically, as well as population acclimatisation to heat waves. The samples sizes for some of the cardiovascular diseases were relatively small when stratified by age group (e.g., cardiac arrest, conductions disorders), and we had limited power to detect interaction effects because of the small number of days that were classified as being heatwaves and having high PM_10_ levels. Therefore, caution is warranted when interpreting the significance of these results. We estimated exposure to heat waves and PM_10_ by calculating the daily city-wide average using monitoring stations, and not by measuring an individual’s personal exposure level, which may have resulted in some exposure misclassification. Our analysis did not account for transfers between episodes of care in the hospital admissions data, and thus it is possible that admissions relating to the same cardiac event for an individual were counted as different events. Further, heat wave forecasts or government-issued heat wave warnings may result in individuals exhibiting avoidance behaviours, especially for people with existing health conditions. This individual level response is beyond the scope of this research.

## 5. Conclusions

This study found some evidence that PM_10_ modifies the association between heat waves and hospital admissions for certain cardiovascular diseases. Our findings, however, showed inconsistencies and largely differed across age group, disease, lag, and heat wave definition. Given the differences found across diseases, our study highlights the need for future studies to consider, where possible, cause-specific outcomes when examining the potential interactive effects of heat waves and ambient air pollution. With both heat waves and levels of ambient particulate matter expected to increase under climate change, it is important to consider potential effect modification by air pollution when examining the impacts of heat waves on cardiovascular morbidity. As our study has shown, this is true even for locations with comparatively low levels of particulate matter, such as Australia. 

## Figures and Tables

**Table 1:** Descriptive statistics for environmental variables in the SSD during the warm season, 2001 to 2013.

Environmental Variables	Mean (SD) Value	Maximum Value	Minimum Value
Weather (Degrees Celsius (°C))			
Daily average maximum temperature	26.40 (4.38)	43.99	14.41
Daily average mean temperature	21.34 (3.05)	32.38	12.47
Daily average minimum temperature	16.27 (2.70)	24.39	6.82
Daily average dew-point temperature	14.92 (3.34)	22.10	−0.13
Ambient Air pollution			
Daily average ozone (pphm)	3.78 (1.48)	11.52	1.04
Daily average PM_10_ (µg/m^3^)	20.43 (11.48)	222.30	4.57
Daily average nitrogen dioxide (pphm)	1.44 (0.59)	4.56	0.29

**Table 2:** Descriptive statistics for EHAs for six cardiovascular diseases in the Sydney Statistical Division (SSD) during the warm season, 2001 to 2013.

	ICD Code (ICD-10-AM)	Total Count	Median (IQR) Daily Value	Maximum Daily Value	Minimum Daily Value
*Cardiovascular Disease*					
Ischemic Heart Disease	I20–I25				
All ages		68,334	37 (31–43)	70	14
0–64 years		28,497	15 (12–19)	34	3
65 years and over		39,837	22 (18–26)	46	5
Heart Failure	I50				
All ages		24,721	13 (10–17)	31	2
0–64 years		3470	2 (1–3)	9	0
65 years and over		21,251	11 (9–14)	28	0
Cardiac Arrest	I46				
All ages		1861	1 (0–2)	6	0
0–64 years		802	0 (0–1)	4	0
65 years and over		1059	0 (0–1)	4	0
Heart Arrhythmia	I47–I49				
All ages		32,682	18 (14–21)	36	5
0–64 years		12,461	7 (5–9)	19	0
65 years and over		20,221	11 (8–14)	25	1
Conduction Disorders	I44–I45				
All ages		3070	1 (1–3)	7	0
0–64 years		641	0 (0–1)	4	0
65 years and over		2429	1 (0–2)	7	0
Hypertensive Diseases	I10–I15				
All ages		3859	2 (1–3)	9	0
0–64 years		1571	1 (0–1)	6	0
65 years and over		2288	1 (0–2)	7	0

**Table 3:** Summary of heat wave characteristics for each heat wave definition used.

Heat Wave Definition	Total Number of Heat Wave Days	Total Number of Heat Wave Events	Average Intensity ^a^ of Heat Wave Day (°C)	Average Duration of Heat Wave (in Days)
HWD01	98	38	35.19	2.58
HWD02	113	43	27.31	2.63
HWD03	114	39	20.75	2.92

^a^ The average intensity was calculated using the temperature metric used in each heat wave definition.

**Table 4:** The effect of heat wave days on EHAs for six cardiovascular diseases on days with high levels of PM_10_ compared to days with low levels of PM_10_ in the SSD during the warm season, 2001 to 2013, for all ages. Effects are presented as odds ratios with their corresponding 95% confidence intervals.

	HWD01				HWD02				HWD03			
	Lag_0_		Lag_1_		Lag_0_		Lag_1_		Lag_0_		Lag_1_	
	Heat Effect	Heat Effect	Heat Effect	Heat Effect	Heat Effect	Heat Effect	Heat Effect	Heat Effect	Heat Effect	Heat Effect	Heat Effect	Heat Effect
	Low PM_10_	High PM_10_	Low PM_10_	High PM_10_	Low PM_10_	High PM_10_	Low PM_10_	High PM_10_	Low PM_10_	High PM_10_	Low PM_10_	High PM_10_
*Cardiovascular Disease*												
Ischemic Heart	0.92	0.92	0.97	0.94	0.98	0.92	0.98	0.92	1.03	0.95	1.04	1.01
Disease	(0.87, 0.98)	(0.86, 0.98)	(0.92, 1.02)	(0.87, 1.01)	(0.93, 1.03)	(0.86, 0.97)	(0.94, 1.03)	(0.85, 0.99)	(0.98, 1.07)	(0.87, 1.03)	(1.00, 1.08)	(0.93, 1.09)
Heart Failure	0.94	1.00	0.83	0.94	0.99	0.97	0.89	0.94	1.01	1.01	0.95	0.93
	(0.85, 1.04)	(0.90, 1.11)	(0.76, 0.90)	(0.83, 1.06)	(0.90, 1.08)	(0.87, 1.08)	(0.83, 0.95)	(0.83, 1.06)	(0.93, 1.09)	(0.88, 1.16)	(0.89, 1.01)	(0.80, 1.07)
Cardiac Arrest	1.06	0.99	1.05	1.30	0.88	1.22	1.08	1.41	0.93	1.13	1.21	1.24
	(0.73, 1.55)	(0.69, 1.41)	(0.77, 1.41)	(0.86, 1.97)	(0.62, 1.23)	(0.86, 1.74)	(0.84, 1.40)	(0.93, 2.14)	(0.68, 1.26)	(0.70, 1.83)	(0.95, 1.55)	(0.75, 2.07)
Heart Arrhythmia	0.95	1.01	0.99	0.94	0.98	1.02	0.99	0.97	1.00	0.96	1.06	0.96
	(0.86, 1.04)	(0.92, 1.10)	(0.92, 1.06)	(0.84, 1.04)	(0.92, 1.06)	(0.93, 1.12)	(0.93, 1.05)	(0.87, 1.08)	(0.94, 1.07)	(0.85, 1.08)	(1.00, 1.12)	(0.85, 1.09)
Conduction	0.84	1.04	0.91	0.92	0.90	0.96	0.94	0.87	1.11	0.89	0.89	0.86
Disorders	(0.63, 1.12)	(0.77, 1.41)	(0.73, 1.13)	(0.64, 1.33)	(0.71, 1.14)	(0.71, 1.30)	(0.77, 1.13)	(0.60, 1.25)	(0.91, 1.37)	(0.60, 1.31)	(0.74, 1.07)	(0.57, 1.30)
Hypertensive Diseases	0.86	0.87	0.91	0.92	0.88	0.82	0.91	0.91	0.90	1.10	0.86	1.30 *
	(0.65, 1.13)	(0.66, 1.16)	(0.83, 1.25)	(0.65, 1.30)	(0.70, 1.12)	(0.61, 1.09)	(0.76, 1.09)	(0.64, 1.29)	(0.74, 1.10)	(0.75, 1.60)	(0.73, 1.02)	(0.90, 1.89)

* Denotes a statistically significant positive interaction term *p*-value (<0.05).

**Table 5:** The effect of heat wave days on EHAs for six cardiovascular diseases on days with high levels of PM_10_ compared to days with low levels of PM_10_ in the SSD during the warm season, 2001 to 2013, for those aged 0–64 years and 65 years and over. Effects are presented as odds ratios with their corresponding 95% confidence intervals.

	HWD01				HWD02				HWD03			
	Lag_0_		Lag_1_		Lag_0_		Lag_1_		Lag_0_		Lag_1_	
	Heat Effect	Heat Effect	Heat Effect	Heat Effect	Heat Effect	Heat Effect	Heat Effect	Heat Effect	Heat Effect	Heat Effect	Heat Effect	Heat Effect
	Low PM_10_	High PM_10_	Low PM_10_	High PM_10_	Low PM_10_	High PM_10_	Low PM_10_	High PM_10_	Low PM_10_	High PM_10_	Low PM_10_	High PM_10_
*Cardiovascular Disease*												
Ischemic Heart Disease												
0–64 years	0.97	0.94	1.03	1.00	1.02	0.92	1.00	0.94	1.07	1.01	1.08	1.09
	(0.88, 1.07)	(0.85, 1.03)	(0.96, 1.11)	(0.89, 1.11)	(0.94, 1.11)	(0.84, 1.02)	(0.94, 1.07)	(0.84, 1.05)	(1.00, 1.15)	(0.89, 1.14)	(1.02, 1.15)	(0.96, 1.23)
65 years and over	0.89	0.90	0.93	0.89	0.95	0.91	0.97	0.90	0.99	0.90	1.00	0.95
	(0.82, 0.96)	(0.83, 0.98)	(0.87, 0.99)	(0.81, 0.98)	(0.88, 1.01)	(0.84, 0.99)	(0.92, 1.03)	(0.82, 0.99)	(0.93, 1.05)	(0.81, 1.01)	(0.95, 1.06)	(0.85, 1.06)
Heart Failure												
0–64 years	1.05	0.88	0.74	0.90	1.17	0.91	0.85	0.93	1.22	1.32	1.03	0.97
	(0.80, 1.38)	(0.66, 1.17)	(0.59, 0.92)	(0.64, 1.27)	(0.93, 1.47)	(0.68, 1.21)	(0.71, 1.02)	(0.67, 1.31)	(1.001, 1.49)	(0.92, 1.89)	(0.87, 1.23)	(0.66, 1.43)
65 years and over	0.92	1.03	0.84	0.96	0.96	0.98	0.89	0.94	0.97	0.97	0.93	0.92
	(0.83, 1.03)	(0.92, 1.15)	(0.77, 0.92)	(0.83, 1.08)	(0.87, 1.06)	(0.88, 1.10)	(0.83, 0.97)	(0.82, 1.08)	(0.90, 1.06)	(0.83, 1.13)	(0.87, 1.01)	(0.78, 1.07)
Cardiac Arrest												
0–64 years	0.90	0.83	1.31	0.78	0.99	1.14	1.30	1.08	0.96	1.35	1.25	1.35
	(0.52, 1.58)	(0.47, 1.45)	(0.84, 2.04)	(0.40, 1.52)	(0.60, 1.62)	(0.67, 1.97)	(0.89, 1.89)	(0.56, 2.08)	(0.62, 1.49)	(0.68, 2.67)	(0.88, 1.77)	(0.63, 2.90)
65 years and over	1.19	1.11	0.91	1.85 *	0.78	1.28	0.93	1.65 *	0.90	0.96	1.18	1.16
	(0.71, 2.01)	(0.70, 1.78)	(0.62, 1.34)	(1.08, 3.16)	(0.48, 1.25)	(0.80, 2.05)	(0.65, 1.32)	(0.96, 2.84)	(0.58, 1.38)	(0.48, 1.89)	(0.84, 1.67)	(0.59, 2.29)
Heart Arrhythmia												
0–64 years	0.91	0.99	1.13	0.91 **	0.96	1.07	1.07	0.91	0.96	0.95	1.03	0.84
	(0.78, 1.06)	(0.86, 1.14)	(1.01, 1.27)	(0.77, 1.09)	(0.85, 1.05)	(0.93, 1.24)	(0.97, 1.18)	(0.76, 1.08)	(0.86, 1.06)	(0.79, 1.15)	(0.94, 1.13)	(0.69, 1.03)
65 years and over	0.97	1.02	0.91	0.95	0.99	0.99	0.94	1.01	1.03	0.97	1.07	1.05
	(0.86, 1.09)	(0.91, 1.15)	(0.83, 0.998)	(0.83, 1.09)	(0.90, 1.09)	(0.88, 1.11)	(0.87, 1.01)	(0.89, 1.16)	(0.95, 1.12)	(0.83, 1.13)	(1.00, 1.15)	(0.90, 1.23)
Conduction Disorders												
0–64 years	0.80	1.03	1.26	1.80	0.91	1.21	1.24	1.36	1.07	1.22	1.06	1.20
	(0.41, 1.54)	(0.52, 2.02)	(0.77, 2.06)	(0.83, 4.01)	(0.54, 1.53)	(0.63, 2.35)	(0.81, 1.91)	(0.61, 3.04)	(0.67, 1.71)	(0.53, 2.78)	(0.72, 1.57)	(0.52, 2.80)
65 years and over	0.84	1.04	0.83	0.77	0.89	0.89	0.87	0.76	1.12	0.81	0.84	0.77
	(0.61, 1.16)	(0.74, 1.47)	(0.65, 1.07)	(0.51, 1.16)	(0.68, 1.16)	(0.63, 1.27)	(0.70, 1.08)	(0.50, 1.16)	(0.89, 1.41)	(0.52, 1.26)	(0.68, 1.04)	(0.48, 1.24)
Hypertensive Diseases												
0–64 years	0.89	0.96	0.89	0.93	0.91	1.16	1.06	1.22	1.11	1.88	1.08	1.82
	(0.58, 1.36)	(0.62, 1.48)	(0.65, 1.24)	(0.55, 1.56)	(0.63, 1.31)	(0.75, 1.78)	(0.80, 1.39)	(0.73, 2.03)	(0.81, 1.52)	(1.07, 3.28)	(0.83, 1.40)	(1.03, 3.20)
65 years and over	0.84	0.82	1.11	0.92	0.87	0.62	0.82	0.74	0.79	0.74	0.74	1.02
	(0.59, 1.21)	(0.56, 1.19)	(0.86, 1.44)	(0.58, 1.44)	(0.65, 1.18)	(0.42, 0.93)	(0.65, 1.05)	(0.46, 1.18)	(0.61, 1.02)	(0.43, 1.25)	(0.59, 0.92)	(0.62, 1.68)

* Denotes a statistically significant positive interaction term *p*-value (<0.05). ** Denotes a statistically significant negative interaction term *p*-value (<0.05).

## Appendix A

**Table A1:** The effect of heat wave days on “emergency” hospital admissions (EHAs) for six cardiovascular diseases on days with high levels of particulate matter (PM_10_) compared to days with low levels of PM_10_ in the Sydney Statistical Division (SSD) during the warm season, 2001 to 2013, for all ages at lag_2_.

	HWD01		HWD02		HWD03	
*Cardiovascular*	Heat Effect	Heat Effect	Heat Effect	Heat Effect	Heat Effect	Heat Effect
*Disease*	Low PM_10_	High PM_10_	Low PM_10_	High PM_10_	Low PM_10_	High PM_10_
Ischemic Heart	1.02	0.93 **	1.00	0.95	0.99	0.98
Disease	(0.98, 1.07)	(0.85, 1.02)	(0.96, 1.04)	(0.87, 1.03)	(0.96, 1.03)	(0.89, 1.07)
Heart Failure	0.87	0.89	0.92	0.89	0.94	0.91
	(0.81, 0.94)	(0.76, 1.03)	(0.86, 0.99)	(0.76, 1.03)	(0.88, 1.002)	(0.77, 1.07)
Cardiac Arrest	1.13	1.26	1.13	1.37	1.13	0.73
	(0.87, 1.47)	(0.75, 2.12)	(0.89, 1.45)	(0.89, 2.09)	(0.89, 1.43)	(0.40, 1.35)
Heart Arrhythmia	0.98	1.00	1.00	1.04	1.02	1.08
	(0.92, 1.05)	(0.87, 1.14)	(0.95, 1.06)	(0.92, 1.19)	(0.97, 1.08)	(0.94, 1.24)
Conduction Disorders	1.01	0.81	0.92	0.88	0.85	0.90
	(0.82, 1.23)	(0.51, 1.28)	(0.76, 1.11)	(0.57, 1.35)	(0.71, 1.01)	(0.58, 1.39)
Hypertensive Disease	1.02	0.94	1.02	0.91	1.00	1.11
	(0.85, 1.23)	(0.62, 1.41)	(0.86, 1.20)	(0.61, 1.35)	(0.85, 1.17)	(0.73, 1.68)

** Denotes a statistically significant negative interaction term *p*-value (<0.05).

**Table A2:** The effect of heat wave days on EHAs for cardiovascular diseases on days with high levels of PM_10_ compared to days with low levels of PM_10_ in the SSD during the warm season, 2001 to 2013, for those aged 0–64 years and 65 years and over at lag_2_.

	HWD01		HWD02		HWD03	
*Cardiovascular*	Heat Effect	Heat Effect	Heat Effect	Heat Effect	Heat Effect	Heat Effect
*Disease*	Low PM_10_	High PM_10_	Low PM_10_	High PM_10_	Low PM_10_	High PM_10_
Ischemic Heart Disease						
0–64 years	1.07	0.96	1.01	0.95	1.00	1.04
	(1.00, 1.14)	(0.84, 1.11)	(0.95, 1.07)	(0.84, 1.09)	(0.94, 1.06)	(0.86, 1.14)
65 years and over	0.99	0.91	0.99	0.94	0.99	0.93
	(0.94, 1.05)	(0.80, 1.02)	(0.94, 1.05)	(0.84, 1.06)	(0.94, 1.04)	(0.82, 1.06)
Heart Failure						
0–64 years	0.84	0.78	0.90	1.02	0.99	0.92
	(0.69, 1.02)	(0.51, 1.19)	(0.75, 1.07)	(0.69, 1.49)	(0.84, 1.18)	(0.60, 1.40)
65 years and over	0.90	0.90	0.92	0.86	0.93	0.91
	(0.83, 0.98)	(0.75, 1.05)	(0.86, 0.99)	(0.73, 1.02)	(0.87, 0.997)	(0.76, 1.08)
Cardiac Arrest						
0–64 years	1.51	0.76	1.33	0.77	1.17	0.61
	(1.01, 2.26)	(0.31, 1.85)	(0.93, 1.91)	(0.34, 1.74)	(0.83, 1.65)	(0.24, 1.57)
65 years and over	0.92	1.71 *	0.98	1.68	1.10	0.83
	(0.65, 1.31)	(0.89, 3.28)	(0.70, 1.38)	(0.89, 3.18)	(0.80, 1.51)	(0.36, 1.90)
Heart Arrhythmia						
0–64 years	1.05	1.04	1.03	1.00	0.98	0.96
	(0.95, 1.16)	(0.84, 1.29)	(0.94, 1.13)	(0.81, 1.22)	(0.89, 1.07)	(0.76, 1.20)
65 years and over	0.95	0.97	0.99	1.08	1.05	1.16
	(0.87, 1.03)	(0.82, 1.15)	(0.92, 1.07)	(0.91, 1.27)	(0.98, 1.12)	(0.97, 1.39)
Conduction Disorders						
0–64 years	1.09	2.07	1.08	1.80	0.81	1.58
	(0.70, 1.72)	(0.79, 5.42)	(0.72, 1.62)	(0.70, 4.66)	(0.55, 1.18)	(0.59, 4.22)
65 years and over	0.99	0.62	0.88	0.73	0.86	0.78
	(0.79, 1.24)	(0.36, 1.07)	(0.72, 1.09)	(0.45, 1.19)	(0.71, 1.06)	(0.47, 1.27)
Hypertensive Disease						
0–64 years	0.83	1.08	1.06	1.21	1.00	1.64
	(0.62, 1.11)	(0.60, 1.94)	(0.82, 1.38)	(0.69, 2.14)	(0.78, 1.28)	(0.91, 2.98)
65 years and over	1.18	0.77	0.98	0.69	0.99	0.78
	(0.93, 1.50)	(0.43, 1.40)	(0.79, 1.22)	(0.39, 1.21)	(0.81, 1.22)	(0.43, 1.42)

* Denotes a statistically significant positive interaction term *p*-value (<0.05).
